# Prospective GIRF‐based RF phase cycling to reduce eddy current‐induced steady‐state disruption in bSSFP imaging

**DOI:** 10.1002/mrm.28097

**Published:** 2019-11-22

**Authors:** Tom Bruijnen, Bjorn Stemkens, Cornelis Antonius Theodorus van den Berg, Rob Hendrikus Nicolaas Tijssen

**Affiliations:** ^1^ Department of Radiotherapy University Medical Center Utrecht Utrecht The Netherlands; ^2^ Computational Imaging Group for MRI diagnostics and therapy, Centre for Image Sciences University Medical Center Utrecht Utrecht The Netherlands

**Keywords:** bSSFP, eddy current, GIRF, MRI, non‐Cartesian, RF phase cycling

## Abstract

**Purpose:**

To propose an explicit Balanced steady‐state free precession (bSSFP) signal model that predicts eddy current‐induced steady‐state disruptions and to provide a prospective, practical, and general eddy current compensation method.

**Theory and Methods:**

Gradient impulse response functions (GIRF) were used to simulate trajectory‐specific eddy current‐induced phase errors at the end of a repetition block. These phase errors were included in bloch simulations to establish a bSSFP signal model to predict steady‐state disruptions and their corresponding image artifacts. The signal model was embedded in the MR system and used to compensate the phase errors by prospectively modifying the phase cycling scheme of the RF pulse. The signal model and eddy current compensation method were validated in phantom and in vivo experiments. In addition, the signal model was used to analyze pre‐existing eddy current mitigation methods, such as 2D tiny golden angle radial and 3D paired phase encoded Cartesian acquisitions.

**Results:**

The signal model predicted eddy current‐induced image artifacts, with the zeroth‐order GIRF being the primary factor to predict the steady‐state disruption. Prospective RF phase cycling schemes were automatically computed online and considerably reduced eddy current‐induced image artifacts. The signal model provides a direct relationship for the smoothness of k‐space trajectories, which explains the effectiveness of phase encode pairing and tiny golden angle trajectory.

**Conclusions:**

The proposed signal model can accurately predict eddy current‐induced steady‐state disruptions for bSSFP imaging. The signal model can be used to derive the eddy current‐induced phase errors required for trajectory‐specific RF phase cycling schemes, which considerably reduce eddy current‐induced image artifacts.

## 1 INTRODUCTION

Balanced steady‐state free precession (bSSFP) sequences offer the highest signal‐to‐noise ratio (SNR)^1^, ^2^, ^3^ and encode multiple physical parameters into the signal.^4^, ^5^ However, the sequence is prone to eddy current‐induced steady‐state disruptions that can severely compromise image quality or the physical parameter quantification.^6^, ^7^ These eddy currents are a direct consequence of the gradients used for the spatial encoding.^8^, ^9^ In particular, the gradients that change dynamically over repetition blocks disrupt the steady–state (eg phase encode gradient), while the static gradients do not (eg slice–selection gradient). Here a repetition block is defined as the pulse sequence diagram with length of one repetition time. These eddy current effects alter the signal evolution and therefore have to be corrected prospectively. One strategy to reduce the impact of the eddy currents is to select an encoding scheme that smoothly varies the gradient waveforms across sequential repetition blocks. This strategy has been applied to reduce the impact of eddy currents in Cartesian bSSFP imaging using phase encode rearranging,^7^, ^9^, ^10^ phase encode grouping^8^, ^11^ or phase encode averaging.^12^ Similar developments were reported in non‐Cartesian bSSFP imaging that primarily aim to minimize angular increments while maintaining incoherent aliasing properties and robustness to motion artifacts.^13^, ^14^, ^15^

While these smoothly varying encoding schemes are effective at reducing eddy current artifacts, they considerably constrain the k‐space trajectory design parameter space, leading to sub‐optimal encoding efficiency. Further, the effectiveness of these smoothly varying encoding schemes is dependent on sequence parameters, such as the resolution, and therefore do not provide a general solution. A second, and more general, proposed strategy is to annihilate the eddy current effect through partial slice dephasing (through‐slice equilibration).^8^ However, this method requires modification of the slice select gradient and is therefore not applicable to 3D acquisitions. A third proposed method is to monitor the eddy current‐induced magnetic field perturbations during a calibration scan using a dynamic field camera^16^ and to subsequently correct the corresponding phase errors by prospectively inserting small gradients and adjusting the RF phase cycling (RF‐PC) scheme of the excitation pulse.^17^ While these “run‐time” adjustments require only minor sequence modifications and provide a direct and effective compensation method, they require additional hardware and a calibration scan, which considerably reduces the practicality for clinical implementation. From these observations, it is evident that there is a clear need for a deterministic signal model that can relate system‐dependent eddy current properties to sequence specific steady‐state disruptions and subsequently to bSSFP image artifacts. Such a general signal model could be taken into account for numerical or empirical sequence optimization or could be used for the direct compensation method.^17^

Recently, the Gradient impulse response function (GIRF) has been proposed as a comprehensive method to characterize the linear and time‐invariant behavior of the entire gradient system.^18^ This characterization includes the eddy current behavior and therefore we hypothesize that the GIRF should contain all the information required to describe the steady‐state disruptions in bSSFP acquisitions. In this work, we show that these eddy current effects are indeed deterministic and can be predicted given the gradient waveform and the system‐specific GIRF. We propose an explicit bSSFP signal model, based on the GIRF, that predicts the impact of the eddy currents on the steady‐state. First we use this signal model to show that the largest component in the steady‐state disruption originates from the zeroth‐order eddy currents. Second, we show with phantom experiments that the proposed signal model can accurate predict eddy current image artifacts for both Cartesian and non‐Cartesian acquisitions. Third, we revisit the prospective compensation method that adjusts the phase of the excitation pulse and we derive the input for RF‐PC directly from the GIRF. We demonstrate that GIRF‐based RF‐PC counteracts the eddy current effects and therefore reduces steady–state disruptions. Finally, we show that the proposed method works for 2D/3D Cartesian and non‐Cartesian sequences and is in principle applicable to any MRI trajectory.

## 2 THEORY

### 2.1 Eddy currents and bSSFP signal model

bSSFP sequences converge to a steady‐state if the following three conditions are met: (1) TR $\ll T_{2}$; (2) The gradients must be zeroth moment nulled; (3) The total phase accumulation (*ϕ*) due to $B_{0}$‐, gradient waveforms (*G*(*t*)) and RF pulses must be constant over repetition block *n*. Bieri et al. showed that eddy currents can violate condition (4) and therefore disrupt the steady‐state.^3^, ^8^ The effect of steady‐state disruption can be directly related to eddy current‐induced time‐varying magnetic fields that accumulate additional phase Δ*ϕ*(*n*) in the transverse magnetization ($m_{\mathrm{xy}}$). This Δ*ϕ*(*n*) can be decomposed in spatially uniform (0th order), spatially linear varying (1st order) and higher order (nth order) magnetic field components. In this work, we refer to these components as $\Delta B_{0}\left(n,\;t\right)$ that induces $\Delta \phi^{0}\left(n\right)$ and Δ*G*(*n*, *t*) that induce $\Delta \phi^{1}\left(n\right)$ with total phase error $\Delta \phi \left(n,\;r\right)\;=\;\Delta \phi^{0}\left(n\right)\;+\;\Delta \phi^{1}\left(n,\;r\right)$. Fischer et al. showed that higher order field contributions are unlikely to exhibit a considerable effect on these phase errors and therefore they are ignored in the signal model.^17^ Note that $\Delta \phi^{1}\left(n,\;r\right)$ is a function of distance *r* from isocenter. The Δ*ϕ*(*n*, *r*) over the entire repetition block can then be described as Equation (1).

(1) $$\Delta \phi \left(n,\;r\right)=\sum_{ax\in x,\;y,\;z}\gamma \int_{0}^{\mathrm{TR}}\left[\Delta B_{0,\;ax}\left(n,\;t\right)+\Delta G_{\mathrm{ax}}\left(n,\;t\right)\;r\right]\;dt$$

Here *γ* is the gyromagnetic ratio and *ax* are the *x*, *y*, *z* axes of the physical gradient coils. The eddy current‐induced time‐varying magnetic fields $\Delta G_{\mathrm{ax}}$ and $\Delta B_{0,\;ax}$ are a function of the gradient waveforms $G_{\mathrm{ax}}\left(t\right)$. The relationship between these fields and *G*(*t*) can be approximated using the zeroth‐ and first‐order ${\mathrm{GIRF}}^{0,\;1}$.^18^ The zeroth‐order GIRF (${\mathrm{GIRF}}^{0}$) describes the spatially uniform field modulations and the first‐order GIRF (${\mathrm{GIRF}}^{1}$) describes the spatially linear field modulations. The GIRFs can be used to express Equation (1) in terms of the known quantity *G*(*t*) Equation (2).

(2) $$\Delta \phi \left(n,\;r\right)=\sum_{ax\in x,\;y,\;z}\gamma \int_{0}^{\mathrm{TR}}\left[{\mathrm{GIRF}}_{\mathrm{ax}}^{0}*G_{\mathrm{ax}}\left(n,\;t\right)+{\mathrm{GIRF}}_{\mathrm{ax}}^{1}*G_{\mathrm{ax}}\left(n,\;t\right)\;r\right]\;dt$$

**Figure 1:**
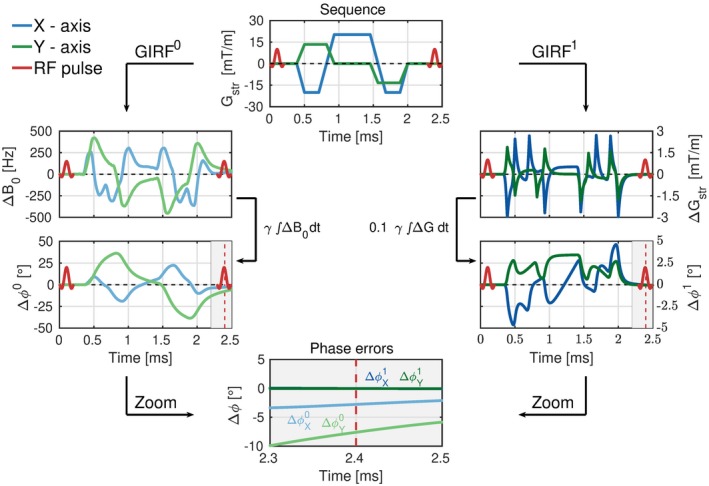
Eddy currents and bSSFP signal model: Top image shows the gradient waveforms corresponding to a typical 2D Cartesian bSSFP acquisition. Left column: The gradient waveform is processed with the ${\mathrm{GIRF}}^{0}$, which induces a field modulation $\Delta B_{0}$ that decays slowly in time. Integrating $\Delta B_{0}$ over time gives the eddy current‐induced phase error $\Delta \phi^{0}$, which is nonzero at the end of the repetition block (red dashed line). Right column: The gradient waveform is processed with the ${\mathrm{GIRF}}^{1}$, which induces a gradient modulation $\Delta G_{\mathrm{str}}$ that decays rapidly in time. Integrating the $\Delta G_{\mathrm{str}}$ at 10 cm off iso‐center gives eddy current‐induced phase error $\Delta \phi^{1}$, which is nonzero at the end of the repetition block (red dashed line). Bottom image: Shows a zoom image of the last 200 μs of the repetition block, which demonstrates the nonzero phase errors. Note that $\Delta \phi^{0}\;\gg \;\Delta \phi^{1}$ for both axes. Note that the blue shades indicate the *X*‐axis and the green shades indicate the *Y*‐axis. Lighter colors indicate the zeroth‐order effects and darker colors indicate the first‐order effects

The process of computing Δ*ϕ*(*n*, *r*) for a standard 2D Cartesian gradient echo sequence is illustrated in Figure 1. Note that Figure 1 shows actually measured field responses where $\Delta \phi^{1}$ is calculated at r = 10 cm (off iso‐center). Equation (2) provides the full description of eddy current‐induced phase accumulation Δ*ϕ*(*n*, *r*), just before the next RF pulse, that could be used to simulate the steady‐state disruption.

### 2.2 Reduced bSSFP signal model

**Figure 2:**
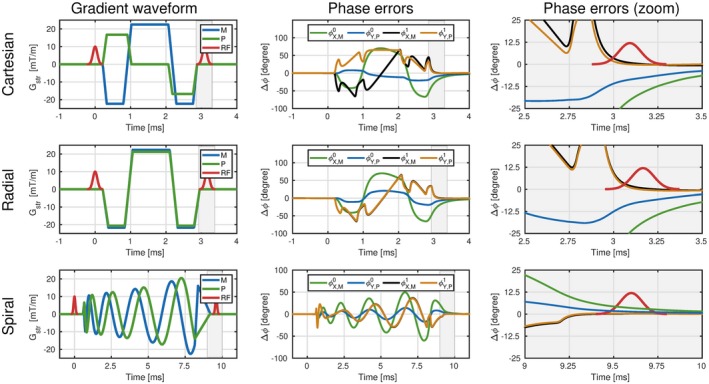
Comparison of zeroth‐order vs first‐order eddy current‐induced phase errors: The three rows represent three different sequences where the RF pulse is positioned as close to the gradient waveform as possible. This setup provides a scenario where the faster decaying first‐order effects could induce large phase errors. Column 1 indicates the investigated gradient waveform. Column 2 represents the corresponding phase errors. Column 3 represents a zoom of the phase error focused on the sequential RF pulse. The phase error at t  = TR (center RF pulse) were for Cartesian: $\Delta \phi_{M,P}^{0}\;=\;\left[-20.2^{\circ },-7.3^{\circ }\right]$ vs $\Delta \phi_{M,\;P}^{1}\;=\;\left[0.9^{\circ },0.0^{\circ }\right]$. Radial: $\Delta \phi_{M,\;P}^{0}\;=\;\left[-18.7^{\circ },\;-6.9^{\circ }\right]$ vs $\Delta \phi_{M,\;P}^{1}\;=\;\left[0.6^{\circ },\;-0.3^{\circ }\right]$. Spiral: $\Delta \phi_{0}^{0}M,P\;=\;\left[3.5^{\circ },\;1.4^{\circ }\right]$ vs $\Delta \phi_{M,\;P}^{1}\;=\;\left[0.2^{\circ },\;0.3^{\circ }\right]$. Here you can observe that $\Delta \phi^{0}\;\gg \;\Delta \phi^{1}$ with an average factor of more than 20. The subscripts in the second column follow the structure of $\Delta \phi_{X,\;M}^{1}$, which correspond to the impact of the *x* gradient coil on the *M* gradient waveform

In Equation (2), the total phase error Δ*ϕ*(*n*, *r*) is dependent on the spatial coordinate *r*, which complicates a straightforward signal model. We observed from system measurements that in general $\Delta \phi^{0}\;\gg \;\Delta \phi^{1}$ holds true for all sequences. Figure 2 provides evidence to support this assumption by showing measured field responses for three sequences. These sequences were selected to have minimal dead‐time between the spatial encoding gradients and the sequential RF pulse to maximize the impact of first‐order effects. A physical explanation to justify $\Delta \phi^{0}\;\gg \;\Delta \phi^{1}$ could be that short lived eddy currents are more prevalent in the first‐order effects, compared to longer lived eddy currents in the zeroth‐order effects. Using this assumption we can simplify Equations (2) and (3).

(3) $$\Delta \phi \left(n\right)=\sum_{ax\in x,\;y,\;z}\gamma \int_{0}^{\mathrm{TR}}\left[{\mathrm{GIRF}}_{\mathrm{ax}}^{0}*G_{\mathrm{ax}}\left(n,\;t\right)\right]\;dt$$

Equation (3) is valid when we consider the repetition blocks individually, but becomes incomplete when we take the sequence history into account. The actual phase accumulation Δ*ϕ*(*n*) at repetition block *n* will also be a function of the (unfinished) phase accumulation during repetition block *n*−1Δ*ϕ*(*n*−1). For clearer notation, we write $\Delta \phi \left(n\right)\;=\;\Delta \phi_{n}\left(n\right)+\Delta \phi_{n-1}\left(n\right)$, where the subscript denotes the repetition block from where the phase errors are generated and the brackets denote the repetition block where the phase errors are evaluated. In particular, for balanced gradient waveforms, the unfinished phase accumulation $\Delta \phi_{n-1}\left(n-1\right)$ will compensate in the repetition block *n* to zero. In other words, $\Delta \phi_{n-1}\left(n-1\right)\;=\;-\Delta \phi_{n-1}\left(n\right)$, where we assume that eddy currents are long enough to induce phase errors in the first block, but short enough to decay within the second block. This compensation of the phase accumulation is related to the linear time‐invariant behavior of the gradient system, where bipolar gradient waveforms induce opposing and time‐delayed phase errors. Therefore, the total phase error in repetition block *n* becomes Equation (4).

(4) $$\Delta \phi_{\mathrm{tot}}\left(n\right)\;=\;\Delta \phi_{n}\left(n\right)-\Delta \phi_{n-1}\left(n\right)$$

Here $\Delta \phi_{\mathrm{tot}}\left(n\right)$ is the total eddy current‐induced phase error experienced by the magnetization, which is induced by gradient waveforms from the previous repetition block $\Delta \phi_{n-1}\left(n\right)$ and the current repetition block $\Delta \phi_{n}\left(n\right)$. Note that this equation directly relates to the concept of using smooth trajectories, which inherently minimize the change of gradient waveforms from TR‐to‐TR ($\frac{\mathrm{dB}}{\mathrm{dTR}}$). Low $\frac{\mathrm{dB}}{\mathrm{dTR}}$ ensures that $\Delta \phi_{n}\left(n\right)-\Delta \phi_{n-1}\left(n\right)\;\approx \;0$ and therefore little phase accumulation occurs. Throughout this work we calculate $\Delta \phi_{\mathrm{tot}}\left(n\right)$ for every repetition block and we incorporate the phase error as additional phase accumulation prior to the next RF pulse.

### 2.3 Prospective GIRF‐based RF phase cycling

**Figure 3:**
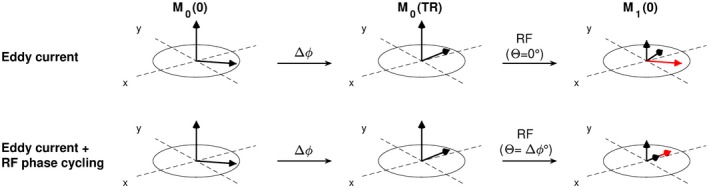
Schematic overview of the prospective RF phase cycling (RF‐PC) method. Top row: Consider the spin ensemble ($M_{0}\left(0\right)$), which experiences $\Delta B_{0}$ field modulations due to eddy currents. The $\Delta B_{0}$ induces phase accumulation Δ*ϕ*, which rotates the transverse magnetization $45^{\circ }$ along the longitudinal axis (*z*) ($M_{0}\left(TR\right)$. This rotation is nonzero when the sequential RF pulse is applied, which misaligns the newly excited longitudinal magnetization (red arrow) with the transverse magnetization ($M_{1}\left(0\right))$ (black arrow). This misalignment propagates over multiple repetition blocks and eventually leads to considerable signal disruptions. Bottom row: Consider the same spin ensemble with the same eddy current‐induced phase accumulation. Now the phase of the RF pulse *Θ* is modified such that the RF pulse aligns the newly excited longitudinal magnetization with the transverse magnetization, therefore restoring the refocusing mechanism

The signal model in Equation (3) assumes that Δ*ϕ*(*n*) is spatially uniform and can accurately be predicted based on the ${\mathrm{GIRF}}^{0}$. These spatially uniform effects can be compensated by adjusting the transmit phase of the sequential RF pulse, ie setting *Θ*(*n*) equal to Δ*ϕ*(*n*).^17^ This adjustment restores the refocusing mechanism of the bSSFP sequences and therefore prevents the disruption of the steady–state. We refer to this method as prospective RF phase cycling (RF‐PC) and the mechanism is illustrated in Figure 3. The RF‐PC scheme then becomes a function of the gradient waveform and can simply be superimposed on conventional phase cycling schemes. Note that RF‐PC is only valid under the instantaneous RF pulse assumption, extension to finite‐length RF pulses would require a frequency modulated RF pulse design to accommodate the varying Δ*ϕ*(*n*, *t*) errors during the pulse. However, basic Bloch simulations showed that the instantaneous RF pulse assumption provides satisfying results for short pulses (<1 ms), which are generally used in bSSFP acquisitions.

## 3 METHODS

GIRF were measured to parameterize the signal model. The signal model was then used to simulate single isochromat steady–state disruptions for varying off‐resonance conditions ($\Delta B_{0}$). These simulations provided insight on how to setup the validation experiments. The first validation experiment included phantom acquisitions, where simulated artifact images were compared to measured artifact images. During these experiments linear shim gradients were applied to emphasize the dependence of the steady–state disruption on $\Delta B_{0}$. The second validation experiments included brain acquisitions, where prospective RF phase cycling was used to reduce eddy current artifacts.

### 3.1 GIRF measurements

To characterize the gradient system, we measured the zeroth‐ and first‐order field responses on a 1.5T MRI (Ingenia, Philips). Twenty‐one triangular gradients with maximum slew rate (180 T/m/s) and varying gradient amplitudes (8.0‐22.5 mT/m) were measured using a 15 cm spherical phantom. The zeroth‐ and first‐order field responses were measured using a variation of the thin slice method.^19^, ^20^, ^21^ A more detailed description of the measurements are reported in Supporting Information I.

### 3.2 bSSFP signal simulations

To investigate the impact of the eddy current‐induced phase errors on the steady–state we computed Δ*ϕ*(*n*) for three different spatial encoding schemes: (1) Linear phase encoding (Lin‐PE); (2) Random phase encoding (Rnd‐PE) and; (3) Golden angle radial (GA‐Rad) encoding. Lin‐PE was selected because of its widespread usage in clinical protocols and robustness to eddy current effects. Rnd‐PE was selected because it resembles the relatively large jumps in k‐space that are commonly seen in highly undersampled acquisitions for compressed sensing,^22^, ^23^, ^24^ low‐high profile ordering for low latency imaging^25^ or *k*−*t* sampling patterns^7^, ^26^ for dynamic imaging. GA‐Rad was selected to represent non‐Cartesian with widespread utility in dynamic imaging.^27^ The Δ*ϕ*(*n*) depend on the sequence parameters and were based on the acquisitions described in Table 1. The maximum Δ*ϕ*(*n*) can be expressed per gradient axes and were $\Delta \phi_{x}\;=\;6.5^{\circ }$ for the Cartesian scans and $\Delta \phi_{x}\;=\;-8.8^{\circ }$/$\Delta \phi_{y}\;=\;-10.2^{\circ }$ for the radial scans. These Δ*ϕ*(*n*) were included in the Bloch model to simulate a single isochromat’s convergence to the steady‐state. The isochromat that was simulated had the following properties: $T_{1}\;=\;1000$ ms, $T_{2}\;=\;80$ ms and $B_{1}\;=\;1.0$. Note that the simulations start in the fully relaxed spin state ($M_{z}\;=\;1$). The simulations were repeated for a range of off‐resonances $\Delta B_{0}\in [-300$ Hz; 300 Hz] to create bSSFP signal profiles.

**Table 1:** Scanner and sequence parameters of the phantom and in vivo experiments

Sequence settings				
	Cartesian phantom	Radial phantom	Cartesian in vivo	Radial in vivo
Field strength	1.5T	1.5T	1.5T	1.5T
Spatial resolution	1.5 × 1.5 × 1.5 mm^3^	1.0 × 1.0 × 5.0 mm^3^	2.5 × 2.5 × 2.5 mm^3^	1.0 × 1.0 × 5.0 mm^3^
Matrix size	100 × 100 × 100	256 × 256 × 1	100 × 100 × 100	256 × 256 × 1
Field‐of‐view	150 × 150 × 150 mm^3^	256 × 256 × 5 mm^3^	250 × 250 × 250 mm^3^	256 × 256 × 5 mm^3^
Repetition time	2.5 ms	3.3 ms	3.4 ms	3.3 ms
Echo time	1.3 ms	1.7 ms	1.7 ms	1.7 ms
Readout bandwidth	1437 Hz/pixel	957 Hz/pixel	478 Hz/pixel	957 Hz/pixel
Number of readouts	10000	402	10000	402
Flip angle	$30^{\circ }$	$30^{\circ }$	$30^{\circ }$	30 $30^{\circ }$

### 3.3 Artifact simulation and experimental validation

To validate the proposed bSSFP signal model, we designed two phantom experiments (Cartesian and Radial encoding) that were compared with simulations. In both experiments, we acquired artifact‐free images using a Lin‐PE bSSFP acquisition and we acquired a $B_{0}$‐map. These data were acquired with a linear shim gradient (1 mT/m) in one direction to highlight the signal dependence on the $\Delta B_{0}$. Both the $B_{0}$‐map and the artifact‐free image were used with the GIRFs to predict the eddy current‐induced image artifacts. The predicted eddy current‐induced artifact images were visually compared with measurements with and without RF‐PC. All experiments were preceded with 5 seconds of dummy TRs to reduce transient state oscillations and all experiments used a short Gaussian shaped RF pulse with time‐bandwidth product  = 2.

#### 3.3.1 Random phase encoded 3D Cartesian acquisition

3D k‐space data were acquired using a random phase encoded (Rnd‐PE) scheme with sequence parameters that facilitate minimal repetition time. Relevant sequence parameters are shown in Table 1. Subsequently the scan was re‐acquired with a random paired phase encoded (Rnd‐P‐PE) scheme, which is known to reduce eddy current‐induced image artifacts.^8^ The acquisitions were repeated using RF‐PC with maximum phase errors of $\Delta \phi_{x}\;=\;-6.5^{\circ }$ / $\Delta \phi_{y}\;=\;-8.1^{\circ }$. Note that the phase errors of all the phase encode lines are a linear combination of $\Delta \phi_{x}$ and $\Delta \phi_{y}$.

#### 3.3.2 Golden angle 2D Radial acquisition

2D k‐space data were acquired using a golden angle radial (GA‐Rad) scheme with sequence parameters that facilitate a minimal repetition time. Relevant sequence parameters are shown in Table 1. Subsequently the scan was re‐acquired with a tiny golden angle (tGA‐Rad) scheme, which is known to reduce eddy current‐induced image artifacts.^14^ The acquisitions were repeated using RF‐PC with maximum phase errors of $\Delta \phi_{x}\;=\;-8.8^{\circ }$ / $\Delta \phi_{y}\;=\;-10.2^{\circ }$. Note that the phase errors corresponding to a specific radial angle is a linear combination of $\Delta \phi_{x}$ and $\Delta \phi_{y}$.

### 3.4 In vivo experiments

This study was approved by the local institutional review board. Following written informed consent, two healthy volunteers were scanned. Three‐dimensional random encoded Cartesian and 2D golden angle radial scans were acquired in the brain with and without RF‐PC. Sequence parameters were equivalent to the phantom experiments (Table 1), besides the linear shim that was disabled. Scans were acquired with volume shimming and $B_{0}$‐maps were acquired to emphasize the dependency of the eddy current artifacts on $B_{0}$. Images were reconstructed on the scanner and visually compared.

## 4 RESULTS

### 4.1 GIRF measurements

**Figure 4:**
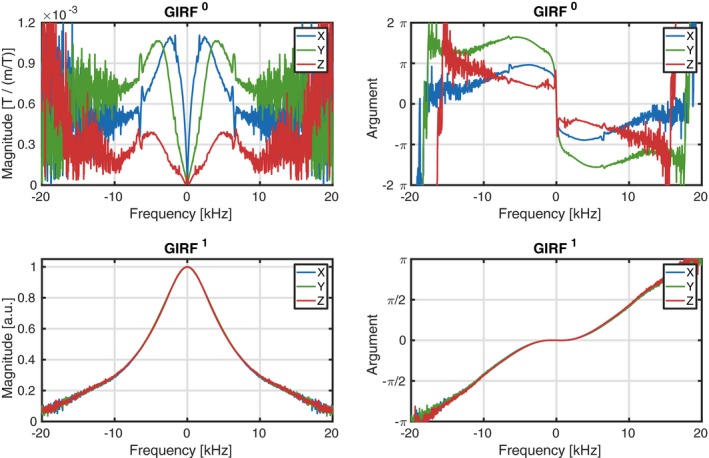
Gradient impulse response functions (GIRF): Top row shows ${\mathrm{GIRF}}^{0}$ with the magnitude on the left and the complex argument on the right. The bottom row shows ${\mathrm{GIRF}}^{1}$ with magnitude on the left and the complex argument on the right

The measured GIRFs are shown in Figure 4. The spectral resolution for the measurements was 33 Hz. The ${\mathrm{GIRF}}^{0}$ show distinct profiles for the three different axes. The ${\mathrm{GIRF}}_{X,Y}^{0}$ show larger magnitudes than the ${\mathrm{GIRF}}_{Z}^{0}$ around the low frequency range, which corresponds to larger eddy current‐induced $\Delta B_{0}$ errors. The ${\mathrm{GIRF}}^{0}$ show distinct peaks around 1780 Hz and 6800 Hz, which could be related to mechanical oscillation frequencies.^18^ Note that the SNR of the measurements was too low for adequate response determination outside the 10 kHz range. The ${\mathrm{GIRF}}^{1}$ show similar behavior for all three axes and have close to zero gradient delay around the low frequencies. The SNR of these measurements was sufficient up to the 20 kHz range.

### 4.2 bSSFP signal simulations

**Figure 5:**
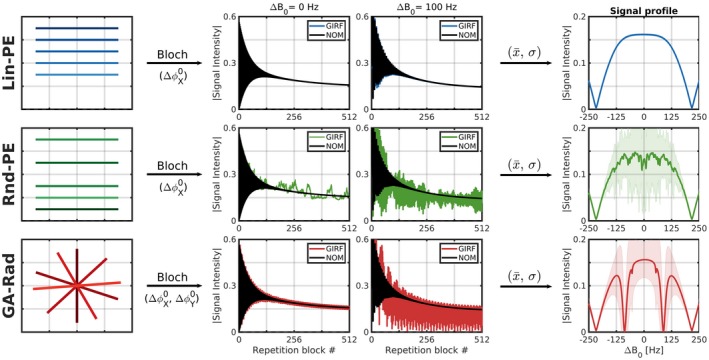
bSSFP bloch simulations in combination with the gradient impulse response functions (GIRF) for three different acquisitions: Top row: Linear phase encoded (Lin‐PE) Cartesian acquisition induces smoothly varying phase errors $\Delta \phi_{0}^{x}\;=\;6.5^{\circ }$, which do not disrupt the steady–state across the entire off‐resonance range. Middle row: Random phase encoded (Rnd‐PE) Cartesian acquisition induces erratic phase errors ($\Delta \phi_{0}^{x}\;=\;6.5^{\circ }$), which considerably disrupt the steady–state across the entire off‐resonance range. Bottom row: Golden angle encoded radial (GA‐Rad) acquisition induces sinusoidal varying phase error ($\Delta \phi_{0}^{x}\;=\;-8.8^{\circ }$ and $\Delta \phi_{0}^{y}\;=\;-10.2^{\circ }$), which disrupt the steady–state across the entire off‐resonance range. At specific off‐resonance frequencies additional zero signal bands occur. Note that the signal profiles represent the magnitude of the mean and the standard deviation of the complex signal intensities over the last 200 repetition blocks in time. NOM  = nominal (without eddy current). GIRF is with eddy current

The impact of the eddy currents corresponding to the three spatial encoding schemes are shown in Figure 5. The first ≈100 readouts show oscillations due to the normal transient behavior of the magnetization. The Lin‐PE Cartesian acquisition showed minor differences from the nominal (default) signal evolution across the entire off‐resonance range. However, the Rnd‐PE Cartesian acquisition showed strong deviations across the entire off‐resonance range. Note that these deviations are erratic and highly coupled to the “randomness” of the encoding pattern. The bSSFP signal profile shows only small deviations in the average signal in time, but shows large standard deviations. The GA‐Rad scheme shows minor magnitude deviations for the on‐resonant case, but shows very large deviations slightly off‐resonance. The bSSFP signal profile shows an additional pair of banding artifacts that are not seen with the other encoding schemes. The position of the bands depend on both the repetition time and the angular increment of the radial acquisition.

### 4.3 Artifact simulation and experimental validation

#### 4.3.1 Random (paired) phase encoded 3D Cartesian acquisitions

**Figure 6:**
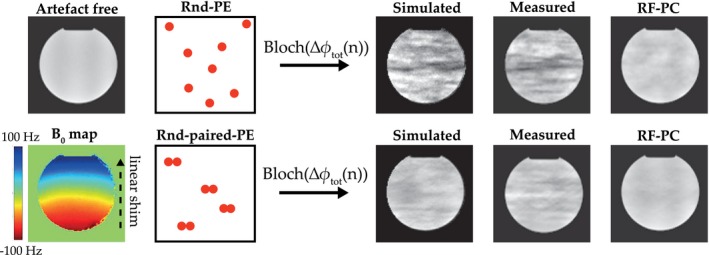
Random (paired) phase encoded 3D Cartesian acquisitions ‐ artefact simulation and experimental validation: First column shows the measured artefact‐free image and the measured $B_{0}$ map. Second column illustrates the 3D Cartesian sampling patterns. Top row of the remaining columns shows the random phase encoded (Rnd‐PE) acquisition and the bottom row shows the random paired phase encoded (Rnd‐paired‐PE) acquisition. Third column shows the simulated artefact images that were based on the artefact‐free image, $B_{0}$ map and the proposed bSSFP signal model. Fourth column shows the measured artefact image. Fifth column shows the measured RF phase‐cycled images

The eddy current‐induced image artifacts induced by the random phase encoded sampling patterns are shown in Figure 6. The simulated Rnd‐PE image closely resembles the measured artifact image. Both the images show a hypo‐intense streak in the center and show large intensity fluctuations from top to bottom. The Rnd‐paired‐PE measured image shows reduced intensity fluctuations compared to the Rnd‐PE. The reduction is also reflected in the simulated image, which is similar to the measured image in magnitude of the intensity fluctuations. RF‐PC considerably reduced these artifacts for the Rnd‐PE as well as Rnd‐paired‐PE acquisitions. Note that both images have small residual artifacts left compared to the artefact‐free image, where the Rnd‐paired‐PE showed the smallest residual artifacts.

#### 4.3.2 (Tiny) golden angle 2D radial acquisitions

**Figure 7:**
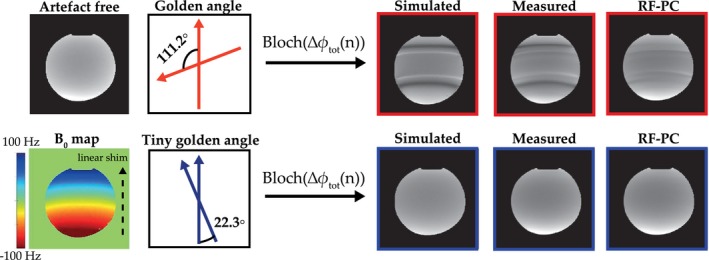
(Tiny) golden angle 2D radial acquisitions ‐ artefact simulation and experimental validation: First column shows the measured artefact‐free image and the measured $B_{0}$ map. Top row of the remaining columns shows the golden angle radial (GA‐Rad) acquisition and the bottom row shows the tiny golden angle (tGA‐Rad) acquisition. Third column shows the simulated artefact images that were based on the artefact‐free image, $B_{0}$ map and the proposed GIRF‐based bSSFP signal model. Fourth column shows the measured artefact image. Fifth column shows the measured RF phase‐cycled images

The eddy current‐induced image artifacts due to the golden angle radial sampling are shown in Figure 7. The simulated GA‐Rad artifact image closely resembles the measured artifact image. Both the images show hypointense and hyperintense lines at the same locations. Note that these lines exactly coincide with the shape of the $B_{0}$‐map and with the locations of the additional bands in the bSSFP signal profile shown in Figure 5. The RF‐PC acquisitions considerably reduced the artifacts, but small residual artifacts remain compared to the artefact‐free image. The simulated tGA‐Rad artifact image and the measured artifact image are almost identical, both show no visual artifacts. The RF‐PC acquisition does not introduce additional artifacts and maintains the image quality.

### 4.4 In vivo experiments—Brain imaging

#### 4.4.1 Random phase encoded 3D Cartesian acquisitions

**Figure 8:**
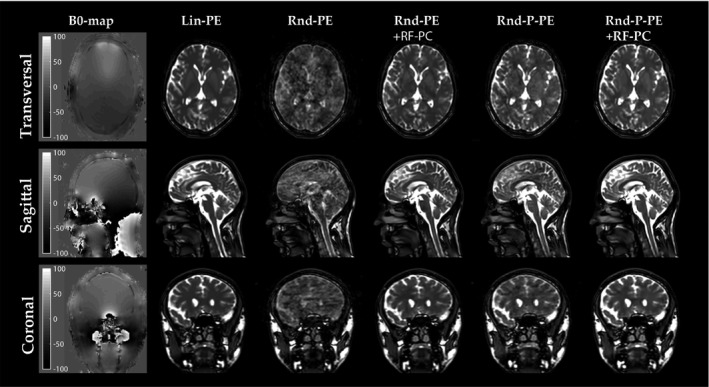
In vivo random phase encoded 3D Cartesian acquisitions. First column shows the $B_{0}$‐map. Second column shows the Cartesian acquisition with linear profile ordering (Lin‐PE). Third column shows the Cartesian acquisition with random phase encode ordering (Rnd‐PE). Fourth column shows Rnd‐PE with RF phase cycling (RF‐PC). Fifth column shows the Cartesian acquisition with random paired phase encode ordering (Rnd‐P‐PE). Last column shows Rnd‐P‐PE with RF‐PC. Sequence parameters are shown in Table 1

Three‐dimensional brain scans were acquired in a healthy volunteer that include a $B_{0}$‐map, Lin‐PE, Rnd‐PE with/without RF‐PC and Rnd‐P‐PE with/without RF‐PC (Figure 8). The $B_{0}$‐map shows that large field inhomogeneity that is typically seen around the tissue/air interfaces. The Rnd‐PE acquisition shows large image artifacts that completely obscure the image structures in the brain. Repeating the acquisition with RF‐PC considerably reduces these artifacts. Note that small residual artifacts are still visible. The Rnd‐P‐PE acquisition shows less image artifacts then the Rnd‐PE acquisition. Repeating the acquisition with RF‐PC further reduces the image artifacts and the image appears similar to the Lin‐PE acquisition.

#### 4.4.2 Golden angle 2D radial acquisitions

**Figure 9:**
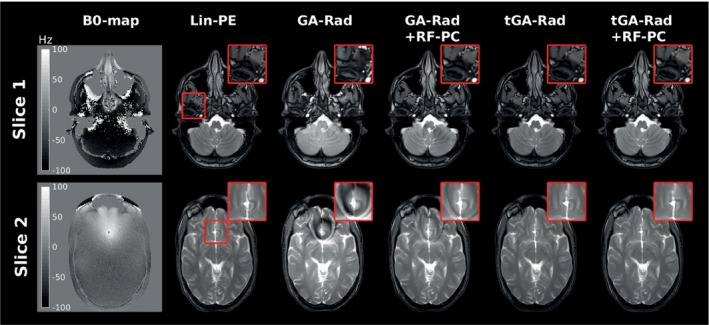
In vivo 2D golden angle radial acquisitions. Two slices were acquired with a $B_{0}$‐map, artefact‐free reference (Lin‐PE), golden angle radial (GA‐RAD), golden angle radial with RF phase cycling (GA‐RAD‐RFPC), tiny golden angle radial (tGA‐RAD) and tiny golden angle radial with RF phase cycling (tGA‐RAD‐RFPC). In both slices the GA‐RAD shows eddy current‐induced image artefacts that are considerably reduced after RF phase cycling. Tiny golden angle images show no deviations from the reference image and adding RF phase cycling does not introduce new artefacts

Two slices were examined using six acquisitions that include a $B_{0}$‐map, Lin‐PE, GA‐RAD, GA‐RAD‐RFPC,tGA‐RAD and tGA‐RAD‐RFPC (Figure 9). The first slice shows large field inhomogeneity around the auditory canals, which leads to eddy current‐induced image artifacts around these areas in the GA‐RAD image. These artifacts include hypo‐and‐hyper‐intense regions, which are clearly visible in the zoom image. These artifacts are not present in the tGA and Lin‐PE images and are considerably reduced in the GA‐RAD‐RFPC image. The second slice shows large field inhomogeneity’s around the frontal lobe, which leads to eddy current‐induced image artifacts in the GA‐RAD image. These artifacts include dark and bright tight bands with curvature similar to the $B_{0}$‐map. These artifacts are not present in the tGA and Lin‐PE images and are considerably reduced in the GA‐RAD‐RFPC image, but residual artifacts remain. Note that there are subtle differences in image contrast between lin‐PE and the GA‐Rad/tGA‐Rad acquisitions. These differences are presumably related to off‐resonance effects or small k‐space trajectory errors in the reconstruction.

## 5 DISCUSSION

In this study, we used the GIRF to formulate an explicit bSSFP signal model that predicts the impact of eddy currents on the steady–state. In particular, we showed that the zeroth‐order impulse response function is the primary factor to describe the impact of eddy currents on the steady–state, while the first‐order impulse response functions play only a minor role. The proposed signal model was validated using computer simulations and experimental imaging which showed good correspondence. Secondly, we revisited a prospective eddy current compensation method that uses RF phase cycling (RF‐PC) schemes to reduce the steady–state disruptions. We showed that RF‐PC is viable without additional field monitoring hardware by using the GIRF. The GIRF were used in combination with the spatial encoding gradients to prescribe RF phase cycling schemes that can easily be computed on the fly. The proposed RF‐PC method does not require any pre‐scans or significant sequence modifications and is in principle applicable to any MRI examination.

The proposed bSSFP signal model provides insight into the effectiveness of several pre‐existing methods to mitigate eddy currents effects. First, we consider the phase encode pairing method proposed by Bieri et al., in which the method mitigated eddy currents artifacts in a wide off‐resonance range for relatively small phase errors (Δ*ϕ*).^8^ In this work, we verified their findings that phase encode pairing reduces the eddy current effects; however, for larger phase errors considerable residual artifacts remain (Figure 6).^17^ In^8^ an explanation was given for the effectiveness of the pairing method, which was that the default $\left[0\;180^{\circ }\right]$ bSSFP phase cycling scheme cancels out two sequential near identical phase errors. Our proposed signal model provides an alternative explanation for the effectiveness of the phase encode pairing, which is related to the approximation of the eddy current behavior as a linear time‐invariant system. The approximation implies that the total phase error in repetition block *n* is the contribution of the current repetition block (*n*) minus the contribution of the previous repetition block (*n*−1), ie ($\Delta \phi \left(n\right)\;=\;\Delta \phi_{n}\left(n\right)-\Delta \phi_{n-1}\left(n\right)$. From this observation it is apparent that phase encode pairing would make the total phase error zero every other repetition block, therefore considerably reducing the steady–state disruption. The second pre‐existing method we consider is the use of tiny golden angles in radial sampling. The effectiveness of the tiny golden angles can be explained using the same observation as for the paired phase encoding. The tiny golden angles induce smoother changes in Δ*ϕ*(*n*) than the golden angle and therefore induce a smaller $\Delta \phi_{\mathrm{tot}}\left(n+1\right)$. The overall underlying observation is that eddy current‐induced steady–state disruption are minimized if the phase error varies smoothly between sequential repetition blocks. This view of looking at eddy currents has some consequences for the design of sampling patterns in bSSFP. The primary consequence is that smoothness of the sampling patterns should be prioritized over phase encode pairing, because it minimizes $\Delta \phi_{\mathrm{tot}}$ instead of nulling it every other repetition block. Alternatively, sampling patterns could be designed such that these minimize $d\Delta \phi_{\mathrm{tot}}/dn$ instead of $\Delta \phi_{\mathrm{tot}}$. These sequences would yield large, but constant, phase errors which do not disrupt the steady‐state and offer more flexibility in pattern design.

The RF‐PC method provides a general prospective compensation strategy to reduce eddy current‐induced steady–state disruptions in bSSFP imaging. The compensation method is based on the observation that zeroth‐order eddy currents (global) effects were the dominant contributor to the steady–state disruption. However, this observation is only valid for the MR systems that were investigated in this study. Other systems could, for example, exhibit stronger mechanical resonances that could enhance the phase errors of the first‐order eddy currents for specific frequencies. These strong resonances were not observed on our systems and therefore the eddy current effects were considered global. As a consequence of these effects being global, the compensation method only requires minor sequence modification (RF phase adjustments) that does not deteriorate the performance (eg smooth encoding schemes). RF‐PC can therefore be used in conjunction with other methods, enabling more robust artifact suppression. The second advantage of our method is that the specific RF phase cycling schemes can be computed on the fly and is easy to generalize for any MRI acquisition.

The proposed bSSFP signal model and RF‐PC method have several limitations that require discussion. The primary uncertainty in the signal model is coupled to the assumption that gradient system was modeled as a linear time‐invariant system. Previous work showed that the assumption is valid to a certain extent, but may be violated due to for example gradient heating.^28^, ^29^ The second limitation is that we did not perform higher order gradient impulse response measurements, which may induce additional phase errors that we did not account for. However, including the higher order phase errors in the RF‐PC compensation strategy is not straightforward because these errors have heterogeneous spatial distributions. The third limitation is that the RF‐PC method operates under the instantaneous RF pulse assumption, which approximates the real system only for very short RF pulses. In reality, we have a time‐varying phase error Δ*ϕ*(*t*) during the RF pulse that requires frequency modulated RF pulses for exact compensation. All these limitations contributed to the small residual artifacts observable in the RF‐PC images. In the future, we envision that the second and third limitation could be jointly tackled by designing dedicated frequency modulated RF pulses, supplemented by small correction gradients,^17^ that generate the exact spatiotemporal transmit distribution to compensate higher order phase errors. However, implementation of such complex pulse sequences requires dedicated multi‐transmit hardware and is out of scope for this study.

Overall, we believe this work contributes to the general understanding of the impact that eddy currents can play in bSSFP sequences. The proposed RF‐PC method could improve the robustness of bSSFP sequences for clinical usage. In particular, the implementation of non‐Cartesian sequences could benefit greatly from this method, since they in general exhibit less smooth encoding schemes. In addition, the proposed GIRF‐based signal model can be used for numerical sequence optimization or to predict the impact of eddy currents on other sequences such as spoiled SSFP sequences or spin‐echo sequences. In addition, we believe that a rigorous understanding of the impact of eddy current on the signal evolution is crucial for quantitative imaging applications that require precise modeling of the physical MR acquisition.^5^, ^30^

## 6 CONCLUSION

To conclude, the zeroth‐order GIRF is the primary factor to predict eddy current‐induced steady‐state disruption in bSSFP imaging and the severity of this disruption strongly depends on the local off‐resonance frequency. The eddy current‐induced steady‐state fluctuations can be considerably reduced by prospectively adapting the RF phase cycling based on the GIRF. We demonstrated a straightforward implementation of the prospective RF phase cycling method, which we believe could improve the robustness of bSSFP imaging for clinical usage and may have considerable impact on bSSFP‐based quantitative MRI.

## ACKNOWLEDGMENT

This work is part of the research program HTSM with project number 15354, which is (partly) financed by the Netherlands Organization for Scientific Research (NWO).

## Supporting information


**FIGURE S1** Pulse sequence diagram of the thin slice measurement. The illustration displays that all gradients are positioned on the same axes and shows the relative timing
**FIGURE S2** In vivo 3D paired phase encoded Cartesian acquisitions. A, Low readout bandwidth acquisition. B, High readout bandwidth acquisitions. Lin‐PE = linear phase encode, Rnd‐P‐PE = random paired phase encode, RF‐PC = RF phase cycling
**TABLE S1** Scanner and sequence parameters of the high‐resolution in vivo experiments
