# Supplementary material for: Prospective GIRF‐based RF phase cycling to reduce eddy current‐induced steady‐state disruption in bSSFP imaging
Source: Magn Reson Med. 2019 Nov 22;84(1):115–27. doi: 10.1002/mrm.28097 (PMC7154723; doi:10.1002/mrm.28097)
Supplement: Supplementary file 1 — FIGURE S1 Pulse sequence diagram of the thin slice measurement. The illustration displays that all gradients are positioned on the same axes and shows the relative timing FIGURE S2 In vivo 3D paired phase encoded Cartesian acquisitions. A, Low readout bandwidth acquisition. B, High readout bandwidth acquisitions. Lin‐PE = linear phase encode, Rnd‐P‐PE = random paired phase encode, RF‐PC = RF phase cycling TABLE S1 Scanner and sequence parameters of the high‐resolution in vivo experiments [file MRM-84-115-s001.pdf]

Supporting Information I: Gradient impulse response function measurements

Pulse sequence

We implemented a modified version of the thin slice method, as described by Brodsky et al., to measure the zeroth and first order magnetic field responses due to gradients [1, 2]. These sequences consists of a slice selection gradient (G_{ss}), a RF excitation pulse and the gradient waveform of interest (G_I). Both these gradients are positioned on the physical same axis and the sequence is repeated in six different modes of operation. These modes can disable gradients (x), make the polarity positive (+) or make the polarity negative (-). These six modes then include:

1) G_{ss}^{+}; G_I^{x}; (Slice 1)

2) G_{ss}^{-}; G_I^{x}; (Slice 2)

3) G_{ss}^{+}; G_I^{+}; (Slice1)

4) G_{ss}^{+}; G_I^{-}; (Slice 1)

5)G_{ss}^{-}; G_I^{+}; (Slice 2)

6) G_{ss}^{-}; G_I^{-}; (Slice 2)

These experiments were repeated for 21 triangular gradients (G_I) with a fixed slew rate of 180 T/m/s and gradient strength varying from 8 - 20 mT/m [3]. Subsequently, the experiments were repeated for all gradient axes. Note that all scans were acquired with a gradient rewinder to compensate for G_I and all data was acquired with a 12 channel head coil. Relevant sequence parameters for the acquisitions include: slice thickness = 3 mm, slice locations = 20 mm off-isocenter, ADC sampling window = 30 ms, ADC sampling frequency = 300 kHz, number of averages = 76, repetition time = 50 ms, flip angle = 20° and scan time = 478 s per gradient axis. The corresponding pulse sequence diagram is shown in figure-x.

Signal processing

The raw k-space data were extracted from the scanner using Reconframe (Gyrotools, Zurich) in Matlab. Data were coil compressed to a single channel using singular value decomposition [4]. The k-space data were phase aligned by setting the phase of the first sample to zero. Second, the data were averaged using complex averaging followed by phase unwrapping. The baseline measurements (G_{ss}^{*};G_I^{x}) were used to correct the background phase for the other measurements with the same G_{ss}, i.e. mode 3) - 1), 4) - 1), 5) - 2), 6) - 2). For the zeroth order measurements, the measurements with G_I^{-} were subtracted from G_I^{+} to remove phase accumulation from off-resonance and concomitant gradients. Subsequently, measurements from the two slices were averaged to remove first order effects. The residual phase accumulation was attributed the

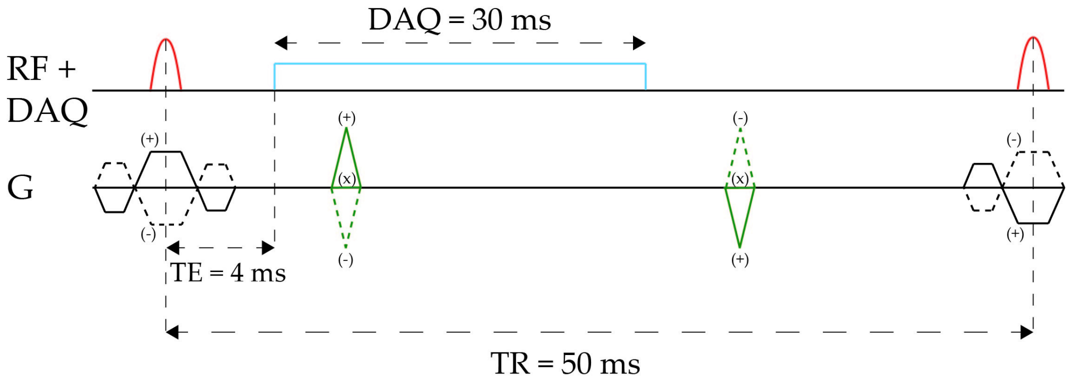

Supporting Information Figure S1: **Pulse sequence diagram of the thin slice measurement.** The illustration displays that all gradients are positioned on the same axes and shows the relative timing.

zeroth order effects and used for the GIRF analysis. For the first order measurements, the measurements with G_I^- were subtracted from G_I^+ to remove phase accumulation from off-resonance and concomitant gradients. Subsequently, measurements from the two slices were subtracted to remove zeroth order effects. The residual phase accumulation was attributed the first order effects and used for the GIRF analysis.

Public dataset

An example of the pulse sequence diagram and the corresponding raw k-space data is made available on the github page provided below. The code includes the gradient measurement signal processing, gradient impulse response computation and bloch simulation steps. https://github.com/tombruijnen/eddy-disruption

Suporting Information II: High resolution Cartesian RF-PC measurements in the lower leg

The experiments described in section 3.4 were repeated with higher spatial resolution in the calf of a volunteer. These results were added to illustrate that the eddy current-induced steady state disruptions vary considerably between acquisitions. These high resolution measurements were done twice (Scan A + B), where the readout bandwidth, and therefore the repetition time, varied between the scans. The sequence parameters are shown in table-S1. The corresponding phase errors predicted with the GIRF were for scan A: $\Delta\phi_y^0 = 0.3°$, $\Delta\phi_z^0 = -4.2°$ and for scan B: $\Delta\phi_y^0 = 0.8°$, $\Delta\phi_z^0 = -7.1°$. The reconstructed images are shown in Figure-S2. The Rnd-P-PE reconstructions show subtle image artefacts in scan A), while these are considerably more apparent in scan B) (also reflected in the difference images). RF-PC removes most of these artefacts, but for scan B) apparent residual artefacts remain.

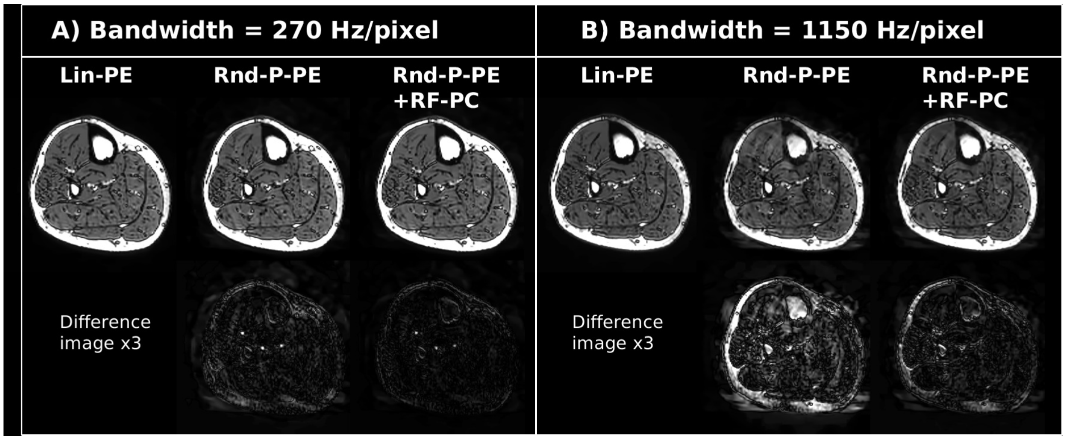

Supporting Information Figure S2: ***In vivo* 3D paired phase encoded Cartesian acquisitions.** A) Low readout bandwidth acquisition. B) High readout bandwidth acquisitions. Lin-PE = linear phase encode, Rnd-P-PE = random paired phase encode, RF-PC = RF phase cycling.

Supporting Information Table S1: **Scanner and sequence parameters of the high resolution *in vivo* experiments.**

**Sequence settings**		
	**Scan A)**	**Scan B)**
Field strength	1.5T	1.5T
Spatial resolution	1.2x1.2x2.3 mm^3	1.2x1.2x2.3 mm^3
Matrix size	133x133x133	133x133x133
Field-of-view	160x160x307 mm ^{3}	160x160x307 mm ^{3}
Repetition time	5.4 ms	3.0 ms
Echo time	2.7 ms	1.5 ms
Readout bandwidth	271 Hz/pixel	1155 Hz/pixel
Number of readouts	18000	18000
Flip angle	30°	30°

References

[1] Brodsky EK, Klaers JL, Samsonov AA, Kijowski R, Block WF. Rapid measurement and correction of phase errors from b0 eddy currents: Impact on image quality for non-cartesian imaging. Magnetic Resonance in Medicine 2013; .

[2] Duyn JH, Yang Y, Frank JA, Van Der Veen JW. Simple correction method for k-space trajectory deviations in mri. Journal of Magnetic Resonance 1998; .

[3] Vannesjo SJ, Haeberlin M, Kasper L, Pavan M, Wilm BJ, Barmet C, Pruessmann KP. Gradient system characterization by impulse response measurements with a dynamic field camera. Magnetic Resonance in Medicine 2013; pp. 583–593.

[4] Buehrer M, Pruessmann KP, Boesiger P, Kozerke S. Array compression for mri with large coil arrays. Magnetic Resonance in Medicine 2007; .
